# Pneumomediastinum as a Complication of Cocaine Insufflation

**DOI:** 10.7759/cureus.38392

**Published:** 2023-05-01

**Authors:** Guarina Molina, Rafael Contreras, Kyle Coombes, Matthew Barbery

**Affiliations:** 1 Internal Medicine, Danbury Hospital, Danbury, USA; 2 School of Medicine, American University of the Caribbean, Cupecoy, SXM

**Keywords:** drug addiction, illicit drugs, cardiopulmonary, pneumomediastinum, cocaine

## Abstract

A 25-year-old man presents to the ED for unresponsiveness after consuming cocaine and other unknown substances. Chest imaging from the presentation was unremarkable, but after developing fever and leukocytosis, he underwent extensive work-up in search of infectious foci. A CT scan of the chest showed a small pneumomediastinum and the possibility of an esophageal tear. After recovering consciousness and the ability to recount events, the patient admitted to the concomitant use of cocaine and opiates via insufflation.

## Introduction

Pneumomediastinum is defined as the presence of air in the mediastinum, which can result from trauma, primary lung diseases, or iatrogenic. In our case, the presumed cause relates to the Valsalva maneuver used to enhance the effects of the drug. This is a rare entity, with a prevalence of 0.002%, and is most common in young males [1]. We present the case of a young man with pneumomediastinum associated with opioids and cocaine inhalation, as well as diagnostic work-up, management, and a review of the literature.

## Case presentation

A 25-year-old male without medical conditions presents for unresponsiveness after insufflating heroin and cocaine. He was brought into the ED after his mother called in a wellness check and was found unconscious at home covered in vomit and urine. He was admitted to the medicine service for the management of encephalopathy in the setting of drug intoxication.

**Figure 1:**
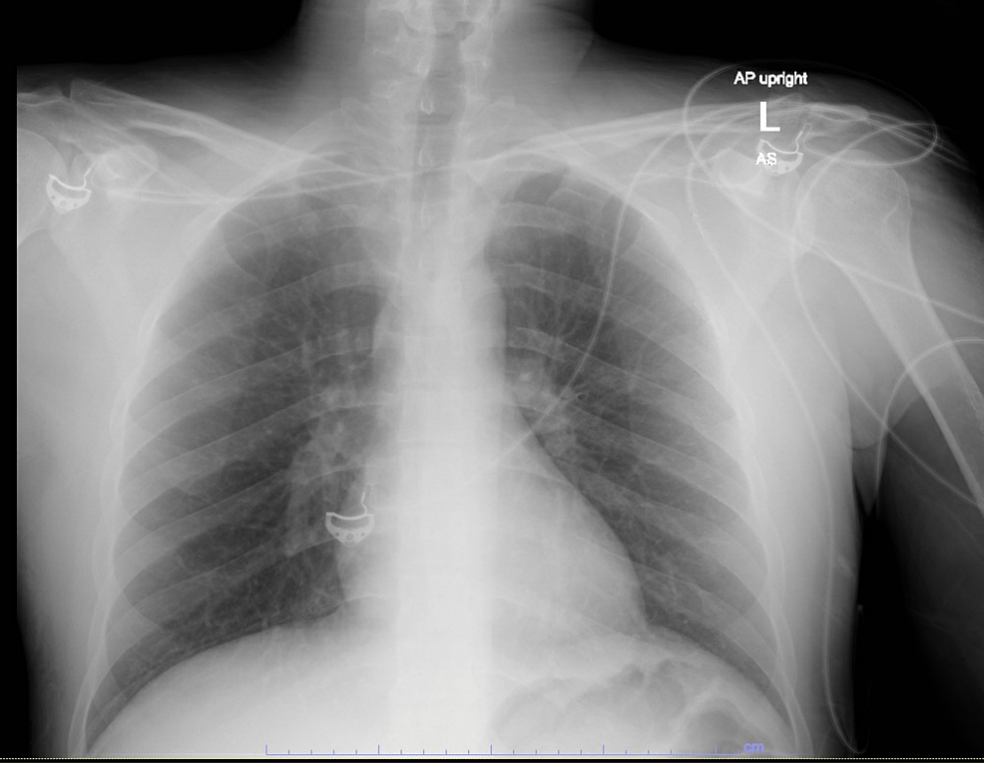
AP chest X-ray from admission

On presentation, he was afebrile, heart rate of 77 bpm, a respiratory rate of 18 breaths per minute with SpO2 of 99%, blood pressure of 153/93 mmHg, with a BMI of 23.8. He was obtunded with a coarse voice, but physical examination, including a cardiopulmonary exam, was unremarkable. There was no evidence of needle track marks. Bloodwork was remarkable for WBC of 12.5 K/uL and lactic acid of 2.5 mmol/L. An antero-posterior (AP) chest X-ray had unremarkable mediastinal contours and no infiltrates or consolidations (Figure 1).

In the first 24 hours of hospitalization, he received supportive treatment for presumed alcohol intoxication and the possibility of withdrawal. He became febrile with a maximum temperature of 100.2 F, which, along with his waning mental status and state from admission, raised concerns of aspiration pneumonia. Infectious blood work, cultures, and urine testing were negative. By the second day, he remained lethargic and attempted to respond to questions with unintelligible responses.

**Figure 2:**
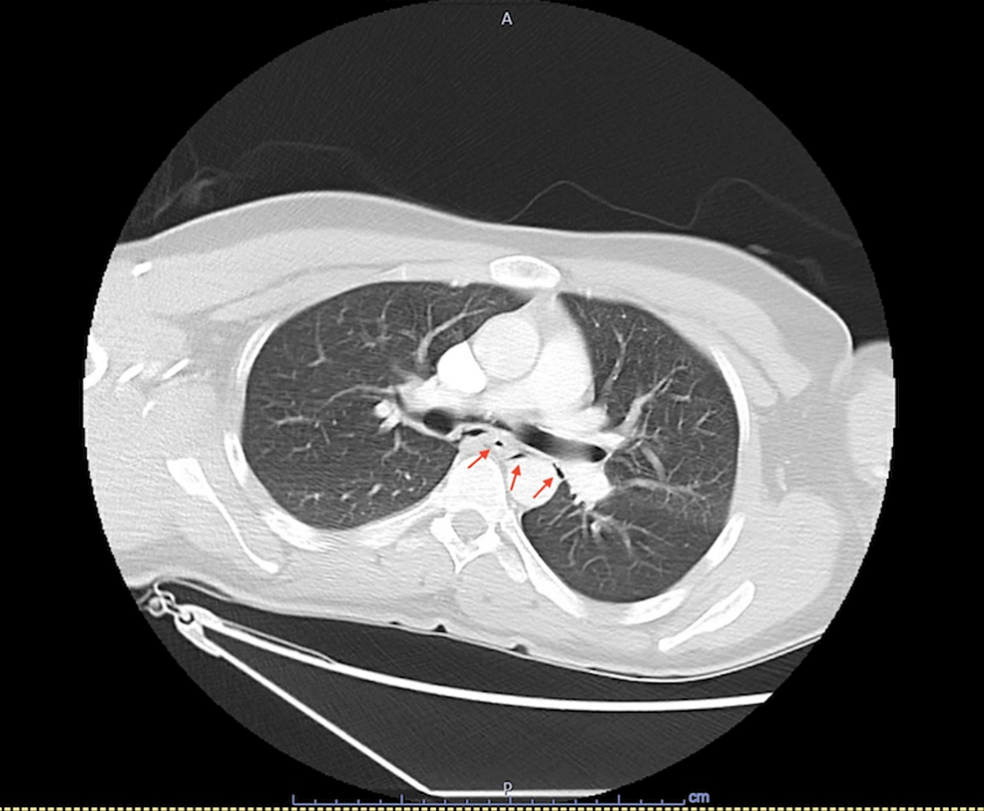
Transverse view of CT abdomen Collections of air surrounding the esophagus (red arrows)

**Figure 3:**
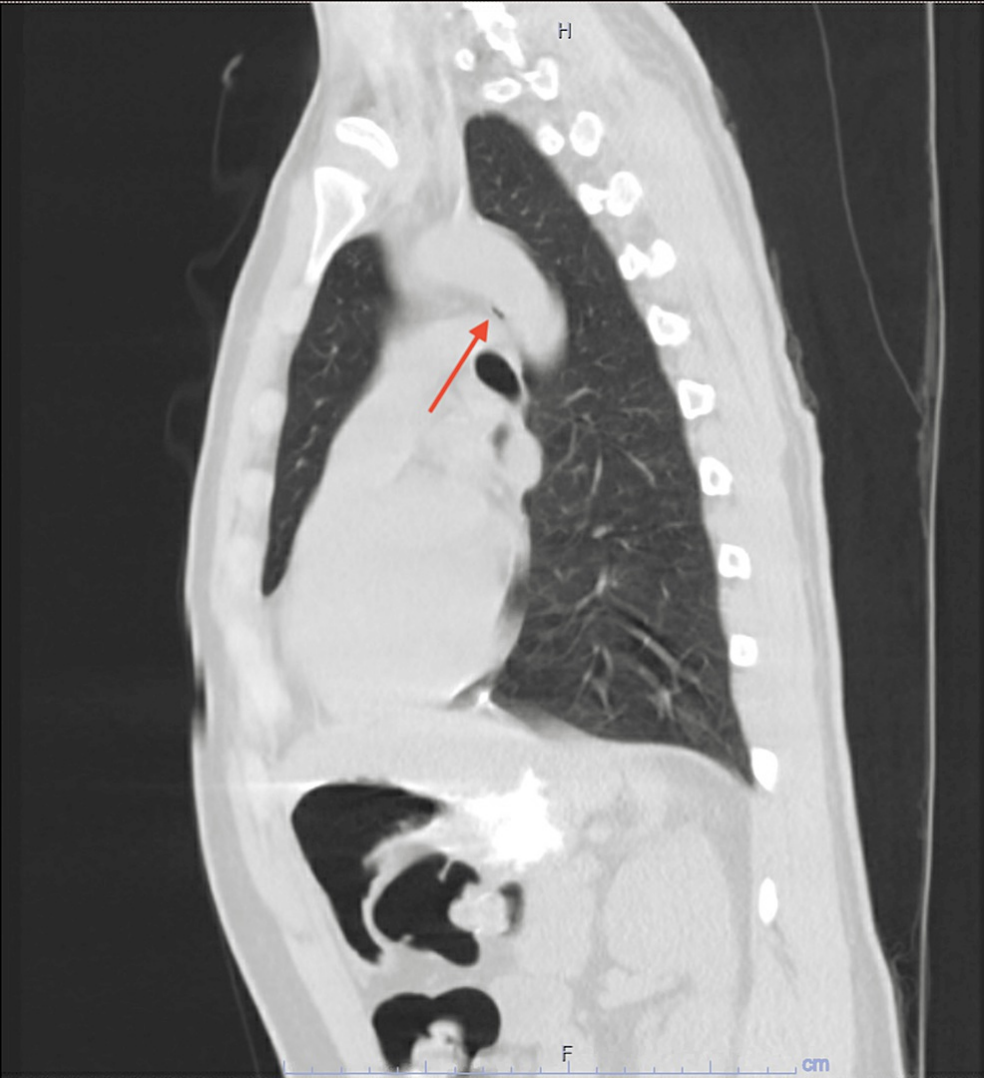
Sagittal view of CT abdomen Collections of air surrounding the esophagus (red arrows)

X-ray of the chest was repeated with no evidence of cardiopulmonary disease or anatomical anomalies. A CT scan of the chest, abdomen, and pelvis with intravenous contrast was performed. It was noted that there was free air (Figures 2 and 3) surrounding the esophagus with concerns of esophageal tear and bilateral parenchymal changes in the base of the lungs. The abdominal and pelvic sections of the imaging were unremarkable.

Due to persistent septic physiology and altered mentation, he was started on empiric broad-spectrum antibiotics: cefepime 1 g IV every 12 hours and metronidazole 500 mg IVPB every 8 hours. After confirming the diagnosis of pneumomediastinum, he was evaluated by a cardiothoracic surgeon who opted for a conservative approach and observation. By hospital day 4, his mentation had returned to baseline, and antibiotic administration was discontinued.

After the sixth day of hospital stay, the patient was discharged home after significant clinical improvement. He was advised to follow up with gastroenterology service within six weeks for endoscopic evaluation. Unfortunately, he was lost to follow up by the medicine service. Five months after this hospitalization, he was admitted to the behavioral health unit for auditory hallucinations. He continues to use cocaine twice a week and heroin every day.

## Discussion

Pneumomediastinum is a rare complication of cocaine use, but several cases with different levels of severity have been described in the literature [2-3]. In addition to the mechanical forces, it has been proposed that cocaine can cause direct toxicity to the lung tissue, leading to alveolar damage and predisposition to rupture. There is a myriad of complications resulting from cocaine and opiate misuse. These include pneumonia (aspiration), acute lung injury, alveolar hemorrhage, pneumomediastinum, aortic dissection, and myocardial infarction [4].

Chest radiography is commonly the first identifying image for pneumomediastinum, with further and more detailed images appreciated in CT. In cases where esophageal involvement is suspected, a barium swallow study or upper endoscopy is suggested. Most of the cases reported have uncomplicated courses, but mediastinitis, pneumothorax, and sepsis can occur.

There are no established guidelines to manage this kind of pneumomediastinum, but most reported cases have been managed conservatively [5] with improvement and resolution when re-evaluated in the outpatient setting.

## Conclusions

Pneumomediastinum is an uncommon manifestation of cocaine misuse and is most prevalent in cases of nasal insufflation. The underlying mechanism involves the Valsalva maneuver meant to enhance the absorption of the drug, causing increased intra-alveolar pressure and rupture. Typical symptoms include chest pain and dyspnea. Our patient presented with altered mentation and a septic profile, with the pneumomediastinum and the esophageal rupture found incidentally as part of the infectious work-up.
